# Identification of cerebrospinal fluid biomarkers for parkinsonism using a proteomics approach

**DOI:** 10.1038/s41531-021-00249-9

**Published:** 2021-11-30

**Authors:** Tainá M. Marques, Anouke van Rumund, Iris Kersten, Ilona B. Bruinsma, Hans J.C.T. Wessels, Jolein Gloerich, Charlotte Kaffa, Rianne A. J. Esselink, Bastiaan R. Bloem, H. Bea Kuiperij, Marcel M. Verbeek

**Affiliations:** 1 Department of Neurology, Donders Institute for Brain, Cognition and Behaviour, Radboud University Medical Center, Nijmegen, The Netherlands; 2 Radboudumc Center of Expertise for Parkinson & Movement Disorders, Nijmegen, The Netherlands; 3 Department of Laboratory Medicine, Radboud Institute for Molecular Life Sciences, Radboud University Medical Center, Nijmegen, The Netherlands; 4 Center for Molecular and Biomolecular Informatics, Radboud University Medical Center, Nijmegen, The Netherlands

**Keywords:** Cerebrospinal fluid proteins, Diagnostic markers

## Abstract

The aim of our study was to investigate cerebrospinal fluid (CSF) tryptic peptide profiles as potential diagnostic biomarkers for the discrimination of parkinsonian disorders. CSF samples were collected from individuals with parkinsonism, who had an uncertain diagnosis at the time of inclusion and who were followed for up to 12 years in a longitudinal study. We performed shotgun proteomics to identify tryptic peptides in CSF of Parkinson’s disease (PD, *n* = 10), multiple system atrophy patients (MSA, *n* = 5) and non-neurological controls (*n* = 10). We validated tryptic peptides with differential levels between PD and MSA using a newly developed selected reaction monitoring (SRM) assay in CSF of PD (*n* = 46), atypical parkinsonism patients (AP; MSA, *n* = 17; Progressive supranuclear palsy; *n* = 8) and non-neurological controls (*n* = 39). We identified 191 tryptic peptides that differed significantly between PD and MSA, of which 34 met our criteria for SRM development. For 14/34 peptides we confirmed differences between PD and AP. These tryptic peptides discriminated PD from AP with moderate-to-high accuracy. Random forest modelling including tryptic peptides plus either clinical assessments or other CSF parameters (neurofilament light chain, phosphorylated tau protein) and age improved the discrimination of PD vs. AP. Our results show that the discovery of tryptic peptides by untargeted and subsequent validation by targeted proteomics is a suitable strategy to identify potential CSF biomarkers for PD versus AP. Furthermore, the tryptic peptides, and corresponding proteins, that we identified as differential biomarkers may increase our current knowledge about the disease-specific pathophysiological mechanisms of parkinsonism.

## Introduction

There is currently no reliable objective test to discriminate Parkinson’s disease (PD) during lifetime from the various forms of atypical parkinsonism (AP), which include multiple system atrophy (MSA), progressive supranuclear palsy (PSP), dementia with Lewy bodies (DLB), corticobasal syndrome (CBS), and vascular parkinsonism. Discrimination of these disorders based on the clinical presentation alone can often be puzzling, especially early in the disease course when symptoms overlap across the different parkinsonism conditions. The clinical diagnosis is based on the most recent specific criteria defined for each disease and includes clinical features, imaging, rate of disease progression, and response to dopaminergic medication^1–6^. However, many of these symptoms have not developed fully in early disease stages, explaining why the rate of misdiagnosis could be up to 20%, even in the hands of movement disorders experts^7^. Therefore, especially in early disease stages, reliable biomarkers are needed for accurate differentiation between PD and AP. Such a timely distinction is important, e.g., for patient counselling since the forms of AP usually have a faster disease progression than PD, with little or no clinical response to levodopa medication. Being able to reliably separate the different parkinsonian syndromes at the earliest possible stage is also critically important for research purposes, allowing the correct patients to be recruited into trials.

Cerebrospinal fluid (CSF) is a rich source for the identification of potential fluid biomarkers for neurodegenerative disorders due to its close proximity to the brain. The CSF composition may directly reflect pathological changes in the brain. Although several studies have identified potential biomarkers for parkinsonian syndromes, none have yet been implemented in clinical practice. So far, quantification of α-synuclein (α-syn) by real-time quaking-induced conversion (RT-QuIC) proved very useful to discriminate parkinsonian disorders with an underlying α-synucleinopathy, such as PD, DLB, and MSA, compared to other types of proteinopathies, such as the tauopathies PSP and CBS^8,9^. However, this assay could not discriminate PD from MSA or DLB. Quantification of neurofilament light chain (NfL) in either CSF or blood may discriminate PD from AP^10–12^, but additional biomarkers may help to increase the specificity to discriminate PD from AP.

The aim of this study was to identify proteins, that could assist in the discrimination of PD from AP in relatively early stages of the disease, and assess their diagnostic value in our cohorts. Such biomarkers may alert clinicians for a timely diagnosis of AP which is more rare than that of PD. The identification of proteins in our study was based on tryptic peptide biomarkers, that are produced after enzymatic digestion of CSF proteins with trypsin and enable mass spectrometric analyses. We used non-targeted (shotgun) proteomics for the discovery of protein biomarkers and targeted (selected reaction monitoring; SRM) mass spectrometry (MS) for validation of our findings. We performed our discovery and validation experiments using patients from a unique longitudinal cohort followed up for up to 12 years. Importantly, all participants had an uncertain diagnosis at the time of inclusion, thereby replicating the clinical challenge faced by clinicians to provide a correct diagnosis, i.e., at a phase in the disease process when many clinical symptoms are overlapping and where diagnostic biomarkers could be very useful.

## Results

### CSF proteomic profiling

Using shotgun proteomics, 5,543 tryptic peptides were identified in the PD, MSA, and non-neurological controls groups. Of these 5,543 peptides, 191 peptides had significantly different levels (*p*-value < 0.05) between PD and MSA.

### SRM assay development and validation

For further validation by SRM, we focused on differential tryptic peptides from the comparison of PD vs. MSA, and therefore 34 tryptic peptides were selected from the untargeted discovery study (Table 1) based on the criteria described in the “Methods” section. During method development, two heavy labelled peptides (FPPEETLK and DLGGFDEDAEPR) could not be robustly detected and were excluded. Therefore, our final SRM assay consisted of 32 tryptic peptides, representing 31 different proteins. The SRM assay was robustly validated and all parameters, such as intra- and inter-assay coefficient of variation (CV), sample stability during measurement, and digestion in different days were within our acceptance criteria of a maximum of 20% variation between replicates (see Supplementary Table 1). Results were considered satisfactory and confirmed the stability of the sample preparation and the equipment during measurement days.

**Table 1:** Overview of tryptic peptides with differential expression between PD and MSA in the discovery study.

Peptide sequence	Protein name	Control	PD	MSA	PD vs MSA *p*-value^a^	Ratio PD vs MSA
GFYFSR	Insulin-like growth factor II	5.64E+06 ± 6.45E+06	8.89E+06 ± 8.98E+06	2.79E+07 ± 4.64E+06	0.004	3.1
VQLSEFSPPGSR	Protocadherin Fat 2	1.78E+05 ± 1.27E+05	4.55E+05 ± 4.46E+05	2.06E+05 ± 1.46E+05	0.008	2.2
DDDFTTWTQLAK	Protein O-linked-mannose beta-1.2-N-acetylglucosaminyltransferase 1	3.79E+05 ± 2.80E+05	5.39E+05 ± 2.93E+05	2.56E+05 ± 1.88E+05	0.008	2.1
ALYYDLISSPDIHGTYK	Pigment epithelium-derived factor	1.38E+07 ± 9.88E+06	1.21E+07 ± 7.53E+06	2.35E+07 ± 6.10E+06	0.008	1.9
HVLFGTVGVPEHTYR	Thy-1 membrane glycoprotein	7.85E+05 ± 9.27E+05	5.63E+05 ± 4.25E+05	1.76E+05 ± 1.48E+05	0.013	3.2
FLDTGVVQSDR	Multiple epidermal growth factor-like domains protein 8	1.18E+06 ± 3.74E+05	1.22E+06 ± 7.37E+05	6.32E+05 ± 4.95E+05	0.013	1.9
NVALVSGDTENAK	Extracellular matrix protein 1	1.07E+06 ± 3.80E+05	1.38E+06 ± 3.33E+05	8.43E+05 ± 5.41E+05	0.013	1.6
LALFPDK	Neuroblastoma suppressor of tumorigenicity 1	5.92E+07 ± 2.16E+07	3.14E+07 ± 1.99E+07	7.18E+07 ± 1.80E+07	0.013	2.3
VFNTPEGVPSAPSSLK	Neuronal cell adhesion molecule	4.89E+06 ± 1.93E+06	6.20E+06 ± 2.63E+06	4.01E+06 ± 1.06E+06	0.013	1.5
SFPLSSEHAK	Cadherin-2	1.04E+05 ± 1.24E+05	1.94E+05 ± 1.58E+05	5.47E+04 ± 5.48E+04	0.018	3.6
LTGISDPVTVK	Noelin	1.82E+05 ± 2.04E+05	3.90E+05 ± 3.00E+05	2.02E+05 ± 1.88E+05	0.019	1.9
FEAFEEDR	Seizure 6-like protein 2	4.53E+05 ± 8.52E+05	2.75E+06 ± 3.24E+06	5.48E+05 ± 9.32E+05	0.019	5.0
FLEQELETITIPDLR	Phospholipid transfer protein	1.13E+06 ± 8.54E+05	6.05E+05 ± 1.10E+06	1.25E+06 ± 6.94E+05	0.019	2.1
LSPYVNYQFR	Neurofascin	1.18E+06 ± 4.11E+05	8.67E+05 ± 7.44E+05	1.58E+06 ± 3.22E+05	0.019	1.8
VLEYLNQEK	Secretogranin-2	6.14E+06 ± 3.04E+06	7.93E+06 ± 5.34E+06	4.59E+06 ± 1.19E+06	0.019	1.7
SYLEITPSR	Inter-alpha-trypsin inhibitor heavy chain H5	7.34E+05 ± 3.47E+05	6.72E+05 ± 2.89E+05	1.20E+06 ± 2.57E+05	0.019	1.8
YGFIEGHVVIPR	CD44 antigen	3.84E+06 ± 1.90E+06	2.72E+06 ± 1.98E+06	7.34E+06 ± 2.29E+06	0.019	2.7
VESLEQEAANER	Amyloid-beta precursor protein	6.46E+05 ± 1.80E+06	1.28E+07 ± 1.49E+07	5.64E+05 ± 1.17E+06	0.026	22.7
NLLDLR	SLIT and NTRK-like protein 1	8.09E+04 ± 1.12E+05	4.59E+05 ± 3.48E+05	3.74E+04 ± 3.53E+04	0.028	12.3
LTVFPDGTLEVR	Leucine-rich repeat and immunoglobulin-like domain-containing nogo receptor-interacting protein 1	5.92E+05 ± 4.49E+05	6.86E+05 ± 3.79E+05	3.20E+05 ± 3.20E+05	0.028	2.1
AFQVWSDVTPLR	72 kDa type IV collagenase	2.99E+05 ± 2.96E+05	3.08E+05 ± 1.93E+05	1.50E+05 ± 1.29E+05	0.028	2.1
AVVEVDESGTR	Plasma serine protease inhibitor	2.32E+05 ± 3.13E+05	5.72E+05 ± 3.81E+05	1.89E+05 ± 2.09E+05	0.028	3.0
FPPEETLK	Carboxypeptidase E	1.25E+06 ± 1.13E+06	8.31E+05 ± 9.74E+05	2.06E+06 ± 9.48E+05	0.028	2.5
LQAPVWEFK	Ceroid-lipofuscinosis neuronal protein 5	1.51E+06 ± 6.12E+05	1.10E+06 ± 8.80E+05	2.34E+06 ± 1.44E+05	0.028	2.1
LFEELVR	Pyruvate kinase PKM	6.78E+06 ± 3.51E+06	6.91E+06 ± 1.07E+07	1.18E+07 ± 5.81E+06	0.028	1.7
SQETGDLDVGGLQETDK	Fibulin-1	1.93E+06 ± 2.50E+06	5.05E+06 ± 4.03E+06	2.06E+06 ± 2.03E+06	0.028	2.5
GAAVSNNIVVRPSR	Neuronal cell adhesion molecule	1.15E+06 ± 1.09E+06	1.83E+06 ± 1.12E+06	1.06E+06 ± 9.45E+05	0.028	1.7
SFQTGLFTAAR	Vitamin K-dependent protein S	5.13E+06 ± 1.47E+06	3.81E+06 ± 2.91E+06	7.58E+06 ± 9.33E+05	0.028	2.0
DLGGFDEDAEPR	45 kDa calcium-binding protein	6.98E+04 ± 1.34E+05	1.47E+06 ± 1.26E+06	1.82E+05 ± 3.80E+05	0.030	8.1
VGIPENAPIGTLLLR	Protocadherin gamma-C5	2.25E+05 ± 1.50E+05	1.79E+05 ± 1.58E+05	9.44E+04 ± 7.99E+04	0.040	1.9
FDFNAFR	Mannosyl-oligosaccharide 1.2-alpha-mannosidase IC	7.43E+05 ± 7.37E+05	8.90E+05 ± 1.01E+06	2.57E+06 ± 5.59E+05	0.040	2.9
TFTLLDPK	N-acetylmuramoyl-L-alanine amidase	3.96E+06 ± 5.50E+06	3.04E+06 ± 3.83E+06	1.16E+07 ± 6.44E+06	0.040	3.8
TSDQIHFFFAK	Antithrombin-III	7.63E+06 ± 1.09E+07	8.13E+06 ± 1.43E+07	2.13E+07 ± 9.60E+06	0.040	2.6
TDGAAPNVAPSDVGGGGGR	Contactin-1	7.96E+05 ± 6.87E+05	1.26E+06 ± 7.58E+05	4.69E+05 ± 5.86E+05	0.040	2.7

Values are expressed as mean of arbitrary intensity ± standard deviation.

^a^Analyzed by Mann−Whitney U test. *PD* Parkinson’s disease, *MSA* multiple system atrophy.

### Tryptic peptides levels in CSF from PD, AP, and controls

For group comparisons, we considered MSA and PSP as one group (AP) because of the relatively low number of PSP cases (*n* = 8) in our study. Total protein concentration was higher in the AP group (mean = 579 mg/L) compared to PD (mean = 533 mg/L) and controls (mean = 426 mg/L, *p* < 0.001), due to high total protein levels in the PSP group (see Table 2). Age was positively correlated to the levels of 23/32 peptides in the PD group and to 18/32 peptides in the non-neurological control group, with correlation coefficients ranging from 0.3 to 0.6 (*p* < 0.05). Therefore, age was included as covariate for group comparisons. Clinical assessment of disease severity (Unified Parkinson’s Disease Rating Scale (UPDRS)) positively correlated with 6/32 peptides in the AP group, with correlation coefficients ranging from 0.4 to 0.6 (*p* < 0.05). No significant correlation of (other) clinical parameters with the levels of any of the tryptic peptides in the PD or AP group was observed.

**Table 2:** Group characteristics and clinical parameters of included patients and controls in discovery and validation studies.

	Discovery				Validation				
	Control	PD	MSA	*p*-value^a^	Control	PD	MSA	PSP	*p*-value^a^
*n*	10	10	5		39	46	17	8	
Age at inclusion (years)	58.9 ± 6.8	61.4 ± 7.9	59.4 ± 7.8	*p* = 0.74	58.3 ± 11.0	57.5 ± 10.0	61.6 ± 7.9	67.0 ± 6.6	*p* = 0.06
Sex (men/women)	6/4	7/3	3/2	*p* = 0.88	17/22	30/16	12/5	4/4	*p* = 0.13
Disease duration (months)	NA	36.6 ± 17.4	34.8 ± 16.2	*p* = 0.85	NA	35.5 ± 32.8	29.9 ± 24.4	35.8 ± 17.7	*p* = 0.79
DM (no/yes)	NA	8/2	3/2		NA	32/14	14/3	6/2	
total protein (mg/L)	467.5 ± 66.4	492.2 ± 54.0	442.8 ± 88.2	*p* = 0.40	425.6 ± 211.2	533.2 ± 164.7	510.7 ± 157.6	723.8 ± 366.1	*p* < 0.0001
Baseline									
UPDRS score^b^	NA	*n* = 10 22.3 ± 6.4	*n* = 5 20.6 ± 11.0	*p* = 0.71	NA	*n* = 45 27.3 ± 12.7	*n* = 17 30.2 ± 11.2	*n* = 8 35.3 ± 14.6	*p* = 0.24
ICARS score^b^	NA	*n* = 9 2.2 ± 1.7	*n* = 4 12.5 ± 15.9	*p* = 0.12	NA	*n* = 42 2.8 ± 3.2	*n* = 13 9.5 ± 11.1	*n* = 6 10.7 ± 7.4	*p* = 0.002
MMSE score^b^	NA	*n* = 10 29.3 ± 1.1	*n* = 5 27.2 ± 2.4	*p* = 0.60	NA	*n* = 46 28.3 ± 2.1	*n* = 16 27.9 ± 2.5	*n* = 8 26.0 ± 3.0	*p* = 0.06
Follow-up 3 years									
UPDRS score^b^	NA	*n* = 7 28.1 ± 10.3	*n* = 4 26.2 ± 14.9	*p* = 0.81	NA	*n* = 39 31.0 ± 14.2	*n* = 7 34.0 ± 6.7	*n* = 6 39.8 ± 10.4	*p* = 0.30
ICARS score^b^	NA	*n* = 7 1.6 ± 1.4	*n* = 4 30.0 ± 19.6	*p* = 0.007	NA	*n* = 36 3.4 ± 3.0	*n* = 7 16.7 ± 16.9	*n* = 6 15.3 ± 10.4	*p* = 0.001
MMSE score^b^	NA	*n* = 8 29.1 ± 1.1	*n* = 4 26.5 ± 2.9	*p* = 0.09	NA	*n* = 36 27.9 ± 2.8	*n* = 7 27.3 ± 2.0	*n* = 6 25.0 ± 4.0	*p* = 0.036

Values are expressed as mean ± standard deviation.

^a^Analyzed by Kruskal−Wallis with Dunn’s multiple correction test or for comparisons between two groups by Mann−Whitney U test, except for sex, which was analyzed using chi-squared test.

^b^Number of available values indicated. *n* number of samples, *DM* intake of dopaminergic medication at CSF collection, *PD* Parkinson’s disease, *MSA* multiple system atrophy, *PSP* progressive supranuclear palsy, *NA* not applicable, *UPDRS* Unified Parkinson’s Disease Rating Scale, *ICARS* International Cooperative Ataxia Rating Scale, *MMSE* mini-mental state examination score.

**Fig. 1:**
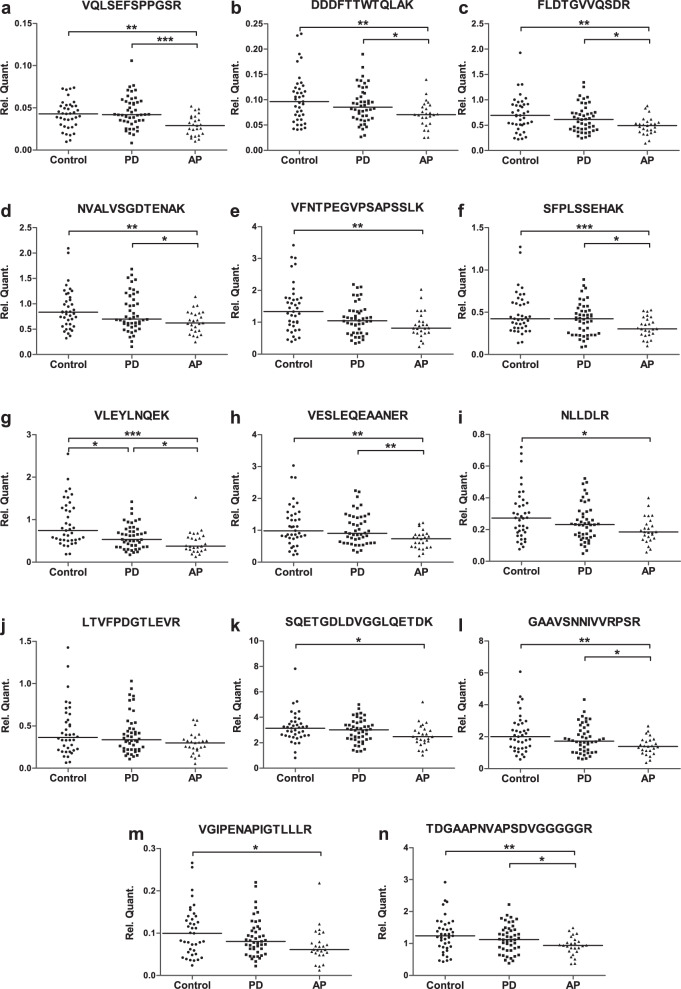
Relative quantification of selected tryptic peptides in cerebrospinal fluid in the validation experiment. Scatter plots of the relative quantification (Rel. Quant.) of 14 selected tryptic peptides in cerebrospinal fluid from non-neurological controls (*n* = 39), and patients with Parkinson’s disease (PD; *n* = 46) and atypical parkinsonism (AP; *n* = 25) in the validation experiment are shown in separate panels (**a**−**n**) for each tryptic peptide, with the peptide sequence plotted on top. Levels of all peptides were lower in AP compared to PD or controls. Statistical significance was based on multiple comparisons including three disease groups, using rank analysis of covariance taking age as a confounding factor, followed by Bonferroni’s multiple correction. Relative peptide levels are shown based on the ratio of endogenous : heavy labelled spiked in peptides, followed by correction for total CSF protein concentration. Bar = median Rel. Quant. value per group; *p < 0.01; **p < 0.001; ***p < 0.0001.

For 14/32 peptides we could confirm our findings from the discovery experiment and replicated the differences in these tryptic peptide levels between PD vs. MSA and PD vs. AP (Table 3 and Fig. 1). The remaining 18/32 peptides did not yield any differences between PD and AP or the observed differences were in the opposite direction as the shotgun experiment. All 14 differential tryptic peptides were present at lower CSF levels in AP compared to both PD and controls, with ratios of PD vs. AP ranging from 1.2 to 1.6. One of these 14 peptides (VLEYLNQEK) also had lower CSF levels in PD compared to controls, while the other 13 tryptic peptides had similar levels in PD and non-neurological controls. Among these 14 peptides, for only 1 peptide (VGIPENAPIGTLLLR) levels were different between men (mean = 0.08) and women (mean = 0.11; *p* = 0.023) in the PD group, but not in other groups. The diagnostic accuracy of the 14 peptides to discriminate PD from AP, i.e., the AUC of the ROC, was moderately high and ranged from 0.60 to 0.76 (Table 3). The strongest potential biomarkers included tryptic peptides belonging to Protocadherin Fat 2, Amyloid-beta precursor protein, Protein O-linked-mannose beta-1,2-N-acetylglucosaminyltransferase 1, and Contactin-1.

**Table 3:** Peptide ratio (endogenous/heavy labelled) for differentially expressed targets between PD and AP in validation study.

Peptide sequence	Protein name	Control	PD	MSA	PSP	PD vs AP vs Control *p*-value^a^	PD vs AP *p*-value^b^	PD vs MSA *p*-value^b^	PD vs AP AUC
VQLSEFSPPGSR	Protocadherin Fat 2	0.04 ± 0.02	0.05 ± 0.02	0.03 ± 0.01	0.03 ± 0.01	< 0.0005^c,d^	< 0.0005	0.001	0.76
DDDFTTWTQLAK	Protein O-linked-mannose beta-1,2-N-acetylglucosaminyltransferase 1	0.10 ± 0.05	0.09 ± 0.04	0.07 ± 0.02	0.07 ± 0.04	0.002^c,d^	0.003	0.009	0.65
FLDTGVVQSDR	Multiple epidermal growth factor-like domains protein 8	0.72 ± 0.35	0.63 ± 0.27	0.49 ± 0.17	0.48 ± 0.19	0.002^c,d^	0.005	0.019	0.64
NVALVSGDTENAK	Extracellular matrix protein 1	0.89 ± 0.40	0.83 ± 0.37	0.62 ± 0.20	0.70 ± 0.27	0.004^c,d^	0.008	0.014	0.63
VFNTPEGVPSAPSSLK	Neuronal cell adhesion molecule	1.45 ± 0.77	1.09 ± 0.48	0.88 ± 0.36	1.00 ± 0.54	0.002^d^	0.048	0.053	0.60
SFPLSSEHAK	Cadherin-2	0.49 ± 0.25	0.42 ± 0.19	0.30 ± 0.10	0.37 ± 0.15	< 0.0005^c,d^	0.004	0.003	0.63
VLEYLNQEK	Secretogranin-2	0.91 ± 0.54	0.60 ± 0.29	0.44 ± 0.18	0.52 ± 0.44	< 0.0005^c,d,e^	0.010	0.026	0.65
VESLEQEAANER	Amyloid-beta precursor protein	1.15 ± 0.66	1.04 ± 0.49	0.74 ± 0.29	0.64 ± 0.24	0.001^c,d^	0.001	0.014	0.69
NLLDLR	SLIT and NTRK-like protein 1	0.29 ± 0.16	0.24 ± 0.12	0.19 ± 0.07	0.21 ± 0.10	0.010^d^	0.032	0.045	0.61
LTVFPDGTLEVR	Leucine-rich repeat and immunoglobulin-like domain-containing nogo receptor-interacting protein 1	0.44 ± 0.30	0.40 ± 0.23	0.31 ± 0.13	0.26 ± 0.12	0.070	0.034	0.147	0.62
SQETGDLDVGGLQETDK	Fibulin-1	3.20 ± 1.19	2.94 ± 0.91	2.64 ± 0.95	2.36 ± 0.82	0.010^d^	0.022	0.093	0.64
GAAVSNNIVVRPSR	Neuronal cell adhesion molecule	2.19 ± 1.18	1.86 ± 0.87	1.41 ± 0.51	1.44 ± 0.69	0.004^c,d^	0.014	0.036	0.64
VGIPENAPIGTLLLR	Protocadherin gamma-C5	0.10 ± 0.06	0.09 ± 0.04	0.07 ± 0.03	0.08 ± 0.07	0.022^d^	0.030	0.065	0.64
TDGAAPNVAPSDVGGGGGR	Contactin-1	1.27 ± 0.56	1.15 ± 0.44	0.93 ± 0.31	0.92 ± 0.28	0.004^c,d^	0.006	0.030	0.65

Values are expressed as mean ± standard deviation.

^a^Analyzed by Rank analysis of covariance, taking age as a covariate, and Bonferroni’s multiple correction test.

^b^Analyzed by Mann−Whitney U test.

^c^PD vs AP.

^d^AP vs Control.

^e^PD vs Control. *PD* Parkinson’s disease, *MSA* multiple system atrophy, *PSP* progressive supranuclear palsy, *AP* atypical parkinsonism (MSA + PSP), *AUC* area under the curve.

### Multi-parametric analysis

We investigated if the 14 tryptic peptides, either in combination with or without other previously established protein biomarkers and clinical data, could improve the discrimination between PD and AP. Four decision trees models were generated by random forest modelling based upon four different datasets containing: (1) the 14 tryptic peptides which were differentially expressed in PD vs. AP, (2) the 14 tryptic peptides and previously identified biochemical markers such as NfL, α-syn, amyloid β42, total tau, phosphorylated tau, and RT-QuIC analysis of misfolded α-syn; (3) the 14 tryptic peptides, the above-mentioned biochemical markers and clinical assessments, such as UPDRS, ICARS, MMSE scores; (4) a combination of the previously identified biochemical markers (as in model 2) with clinical assessments (as in model 3). An overview of the biochemical markers and clinical parameters that were included, next to tryptic peptides, in the models for the discrimination of PD and AP is provided in Supplementary Table 2. The model created with dataset 1 included all 14 peptides and had an AUC of 0.53. The model based on dataset 2 included the peptides VESLEQEAANER (Amyloid-beta precursor protein), VQLSEFSPPGRS (Protocadherin Fat 2), DDDFTTWTQLAK (Protein O-linked-mannose beta-1,2-N-acetylglucosaminyltransferase 1), FLDTGVVQSDR (Multiple epidermal growth factor-like domains protein 8), VFNTPEGVPSAPSSLK (Neuronal cell adhesion molecule), SQETGDLDVGGLQETDK (Fibulin-1) together with CSF levels of NfL, p-tau, and age. This model had an AUC of 0.86. The model of dataset 3 included the peptide SFPLSSEHAK (Cadherin-2) combined with ICARS score, presence or absence of cerebellar dysarthria, disease stage, CSF NfL, orthostatic hypotension score, UPDRS right leg agility score, Hoehn and Yahr score, UPDRS postural stability score, and verbal fluency score. The model also had an AUC of 0.86. The model of dataset 4 included tandem gait score, orthostatic hypotension score, UPDRS scores (bradykinesia, right arm rigidity, right leg agility), verbal fluency score, and CSF levels of NfL. This model had an AUC of 0.88.

## Discussion

In this study, we used untargeted MS to identify tryptic peptides in CSF as potential biomarkers that could discriminate parkinsonian disorders, and performed an independent validation of our findings by targeted MS. For this purpose, we purposely included only patients with clear signs of parkinsonism but an uncertain diagnosis at the time of inclusion and CSF collection, but in whom a silver standard diagnosis was made 3−12 years later based on the rate of progression, response to treatment and possible development of red flags. This approach served to replicate the challenge that clinicians face in everyday clinical practice when a clinical diagnosis has to be established in movement disorders patients with only partially developed clinical syndrome. Under such circumstances, having reliable diagnostic biomarkers would be very helpful.

Both untargeted and targeted MS methods proved to be reliable and robust methods to identify tryptic peptide biomarkers and provided a relative quantification of the levels of these peptides in CSF. We developed a protocol for the evaluation of SRM analysis of tryptic peptides in CSF. The newly developed assay procedure was very robust since it proved to be very stable during several measurement days (CV < 10%), it was reproducible across different sample preparation days, and was resistant to multiple freeze/thaw cycles. Therefore, this SRM assay may be useful for other CSF biomarker studies as well.

The SRM assay confirmed our findings for many tryptic peptides from the discovery experiment, illustrating the robustness of the shotgun proteomics for biomarker identification. For 14 tryptic peptides, we found lower CSF levels in AP compared to non-neurological controls and PD, both in the discovery and validation experiments, and they individually discriminated PD from AP with a diagnostic value up to 76%. Multivariate analysis by random forest modelling did not increase the discriminative value between PD and AP when only peptides were included in the model. The lower discriminative value generated by random forest modelling compared to individual tryptic peptides (53% vs. 76%) could be explained by the low number of variables (14 peptides) included in the analysis, and on top of that, the model was developed in 70% of our cohort and validated in remaining 30%. However, by including more variables, such as other CSF protein biomarkers and/or clinical assessments, the random forest algorithm was capable to provide a better discrimination between disease groups, increasing the accuracy to 86%. Interestingly, very comparable AUC values (0.86−0.88) were obtained for the models 2, 3, and 4, suggesting that a combination of the tryptic peptides identified in the current study with established protein biomarkers (NfL, α-syn, amyloid β42, total tau, phosphorylated tau, and RT-QuIC analysis of misfolded α-syn) has similar additional diagnostic value as clinical data in combination with these established markers. These models including CSF tryptic peptides and clinical assessments offers a great advantage to help clinicians to identify a correct diagnosis of parkinsonian disorders, but need to be tested in independent cohorts.

Aside from a potential role in differential diagnosis, several of the 14 identified tryptic peptides, which are derived from 13 different proteins (Table 3), have a known role in neurodegeneration, which sheds new light on potential disease mechanisms in PD vs. AP. Six out of 13 proteins (Protocadherin Fat 2, Cadherin-2, Protocadherin gamma-C5, Neuronal cell adhesion molecule (2 tryptic peptides), Fibulin-1, Contactin-1) are involved in cell−cell adhesion, an important mechanism of synaptic function maintenance^13^. Two other tryptic peptides/proteins found in our study, SLIT and NTRK-like protein 1^14,15^, and Amyloid-beta precursor protein^16^, also play a role in synaptogenesis. Dysfunctional synapses contribute to neurodegeneration^17^, and dysregulation of these proteins may add to such dysfunction in AP syndromes. A meta-analysis on imaging studies showed that presynaptic dopaminergic function is 34% lower in PSP as compared to PD and MSA^18^. Moreover, in a study published after this meta-analysis, evidence-based on DAT SPECT data was obtained supporting a faster decline of presynaptic function in MSA compared to PD as well^19^. Our findings added several potential molecular biomarkers to this imaging-based evidence of synaptic dysfunction. Studies using immunohistochemistry on brain tissues, animal and in vitro studies may be useful in confirmation of altered expression of the proteins in AP and their localization.

The adhesion protein Cadherin 2 may play a protective role in dopaminergic neurons^20–22^. Loss of Cadherin-2 compromises neuronal differentiation, via the Wnt signalling pathway^23^. Lower levels of Cadherin-2 have previously been found in CSF from PD patients compared to controls^24^. We could, however not replicate this difference in PD vs non-neurological controls, but we did find lower levels in AP vs PD. We could not retrieve any studies investigating the role of Cadherin 2 in MSA or PSP. However, Cadherin 2 is involved in the process of myelination in oligodendrocytes^25,26^, which are the affected neurons in MSA. Lower levels of Cadherin 2 in MSA compared to PD at early disease stage could be involved in the more rapid disease progression of MSA compared to PD, but further studies need to clarify the Cadherin 2 levels in MSA.

Lower CSF levels of the peptide LTVFPDGTLEVR (Leucine-rich repeat and immunoglobulin-like domain-containing nogo receptor-interacting protein 1, LINGO-1) in AP compared to PD could be related to demyelination in MSA as compared to PD. LINGO-1 is a transmembrane protein that negatively regulates oligodendrocyte differentiation and axon myelination^27^. The regulation occurs by inhibition of the RhoA pathway, decreasing the expression of myelin basic protein (MBP)^27^. Functional studies demonstrated the presence of LINGO-1 in dopaminergic neurons and oligodendrocytes^27–29^. A meta-analysis identified LINGO-1 polymorphisms related to decreased risk of PD, but not of MSA^30^. In MSA, accumulation of misfolded α-syn occurs in oligodendrocytes, which are the cells responsible for myelin maintenance. Myelin dysfunction in MSA precedes α-syn accumulation and neuronal loss^31^, therefore myelin dysfunction might be an important early mechanism of neurodegeneration in MSA. In previous studies of our group, we found increased levels of MBP in the CSF of MSA patients compared to PD patients^32,33^. Although the specific mechanism underlying the lower LINGO-1 levels in MSA compared to PD remains unclear, abnormal levels of the peptide LTVFPDGTLEVR may be an indication of early disturbances in oligodendrocyte myelin production in MSA, consistent with the increased CSF MBP levels in MSA.

The peptide VLEYLNQEK (secretogranin-2), was the only peptide in our study, which discriminated PD from both controls and AP. Secretogranin-2 is a protein that is cleaved into peptides and secreted in vesicles, releasing the neuropeptide named secretoneurin, a peptide that stimulates dopamine release in striatal neurons and basal ganglia^34,35^. Therefore, disruptions in secretogranin-2 levels might be related to altered levels of dopamine release in the synaptic cleft. Recently, one study showed co-localization of secretogranin-2 with aggregated α-syn and phosphorylated tau in brain tissue of a PD animal model, suggesting an involvement of these proteins in synaptic trafficking^36^. A previous proteomics study identified lower CSF levels of secretogranin-2 in PD compared to controls^24^, consistent with our results. The secretogranin-2 might be useful as an early biomarker to demonstrate dopamine disturbances in parkinsonian syndromes.

The remaining three identified tryptic peptides were derived from proteins involved in the regulation of cellular communication (Multiple epidermal growth factor-like domains protein 8), extracellular structural function (Extracellular matrix protein 1), and protein glycosylation (Protein O-linked-mannose beta-1,2-N-acetylglucosaminyltransferase 1), with no known relation with neurodegeneration or previous link with parkinsonism.

Several previous studies aimed to discriminate PD from AP by using CSF proteomic profiling. In one study 2,000 (poly)peptides in CSF of PD, AP (MSA, PSP, and CBD), and controls were analyzed using the method of surface-enhanced laser desorption/ionization time-of-flight mass spectrometry (SELDI-TOF MS)^37^. In this study, none of the features could discriminate PD from controls, whereas four proteins or protein fragments (ubiquitin, beta2-microglobulin, and two fragments of secretogranin-1) discriminated either MSA or PSP from PD/controls. Four peptides of secretogranin 1 were identified in our discovery experiment, and at lower levels in MSA compared to PD, confirming these previous findings. However, the peptides belonging to this protein did not qualify for our SRM assay, and therefore we could not confirm it in our validation experiment. In yet another study, using Orbitrap MS, 5,043 protein-derived tryptic peptides were identified in CSF in a discovery cohort of PD, AP (MSA, PSP, and CBS), and controls^38^. The number of peptides is quite comparable to our findings (5,043 vs. 5,543 peptides in our study). In their discovery and validation experiments, up to 90 peptides were detected at significantly lower levels in AP compared to controls (*p* < 0.05), but there were no differences for PD vs. AP or PD vs. controls, as we observed in our study.

Few limitations may apply to our study. First, the long storage time of CSF samples may have affected our results. A previous study investigated the stability of CSF proteins up to 12 years storage on −80 °C^39^, and no differences were found over time. Furthermore, all PD and AP samples in our study were retrieved in the same period, and therefore, we do not expect that storage time is a major factor that may have affected the results of the differential levels in these patients. A second limitation may be related to the final diagnoses of the patients, which were based on clinical assessments and not on neuropathological examinations. However, given the very long follow-up of the patients in our cohorts (up to 12 years), and the independent assessment by two experienced movement disorder specialists, we believe that the rate of misclassification has been reduced to a minimum. Importantly, the long follow-up time allowed us to consider the rate of progression, response to therapy, and development of any red flags into the diagnostic process. We also included brain imaging findings in the diagnostic process. Based on these clinical parameters, a reliable ‘silver standard’ diagnosis can be made in most patients. Third, for 18 tryptic peptides, selected from our discovery experiment for validation, the results did not match in both experiments, which reinforces the need of robust independent validation studies before conclusions can be reached, which applied to the remaining 14 tryptic peptides. Fourth, apart from MSA, we only had a limited number of other cases with other causes of AP, such as PSP, CBD, or DLB, in our validation study, due to the relative low representation of these patients, for which also CSF was available, from our longitudinal cohort. Since AP comprises a heterogeneous group of disorders, including both synucleinopathies and tauopathies, the small number of AP cases other than MSA also limits the translation of our findings to potential disease mechanisms in e.g., PSP, and will probably mainly reflect changes in MSA.

One of the strongest aspects of our study is the use of two independent cohorts of patients for discovery and validation. In addition to that, we also performed our validation using a different MS technique (SRM) than in the discovery, and we confirmed the consistency of 14 tryptic peptides to discriminate PD from AP. A second strong point is the unique longitudinal study, in which patients were initially included with clear parkinsonian symptoms, but with an uncertain diagnosis at baseline, i.e., at a time in the diagnostic process where fluid biomarkers are needed most. As such, our cohort offers excellent opportunities for fluid biomarker discovery and validation, as we demonstrate here. Besides providing new insights for potential biomarkers to help clinicians to discriminate parkinsonian disorders, this may also provide new insights into differences in the underlying pathophysiological processes for PD as compared to AP.

In summary, proteomics is a powerful tool to identify peptides in CSF for discrimination of parkinsonian disorders. Our newly developed SRM assay proved to be very robust and offered a reliable relative quantification of tryptic peptides in CSF. Our validation experiment confirmed the potential of 14 CSF peptides to discriminate PD from AP, already at an early disease stage when there is still a high level of uncertainty about the underlying aetiology of the specific movement disorder. The discriminative value of these tryptic peptides could be enlarged by the combination with existing biochemical markers or clinical assessments. Finally, our study may provide new insights into the underlying pathophysiological processes of each disorder.

## Methods

### Patients and samples

For both the discovery and validation experiments, we included participants from a longitudinal study^40^, who all had clear clinical signs of parkinsonism, but with an yet unclear diagnosis at the time of inclusion, and who had been recruited from our movement disorders outpatient clinic between January 2003 and December 2006 at the Radboud University Medical Centre (Nijmegen, the Netherlands). In total, 25 CSF samples were included in the discovery experiment (PD, *n* = 10; MSA, *n* = 5; non-neurological controls, *n* = 10). For validation of our initial findings of the discovery phase we used 110 CSF samples from PD (*n* = 46), MSA (*n* = 17), PSP (*n* = 8), and non-neurological controls (*n* = 39).

At the time of inclusion, patients underwent a structured standardized neurologic examination by movement disorders specialists. Lumbar puncture, was performed within 6 weeks after the initial visit. The design of this study, methodology, and patient inclusion have been extensively described^40^. After three and 12 years of inclusion, the diagnosis of all participants was critically revised again and a silver standard clinical diagnosis was established by two independent movement disorder specialists. To establish this diagnosis, the clinical experts used the most recent clinical criteria at that time^1–6,41,42^, combined with the now available long-term response to therapy, the rate of disease progression, and the possible development of red flags, which may alert clinicians to an alternative diagnosis. Patient characteristics are presented in Table 1. For correlations of the newly identified biomarkers from the present study with other, established protein biomarkers, we used previously published data on NfL, α-syn, total tau, phosphorylated tau, amyloid-β42, and α-syn RT-QuIC^8,12,40,43,44^. For details on the assays used for quantification of these protein biomarkers, see Supplementary Methods.

Clinical assessments at baseline and after 3 and 12 years of follow-up included the Hoehn and Yahr scores^45^, Unified Parkinson’s Disease Rating Scale (UPDRS)^46,47^, Mini-Mental State Examination (MMSE)^48^, and International Cooperative Ataxia Rating Scale (ICARS)^49^.

For comparison, we selected a group of non-neurological control patients who had underwent a lumbar puncture because of a suspected central neurological disorder. All selected control cases were free of neurological disease, as determined after careful examinations. Moreover, their CSF composition, such as leukocyte and erythrocyte count, glucose, blood pigments, lactate, and (if assessed) oligoclonal immunoglobulin G bands were all within the reference ranges for their age group.

All CSF samples included in this study were collected in polypropylene tubes, centrifuged at 800 × *g*, aliquoted, and stored in polypropylene tubes at −80 °C until use. All patients with PD or AP provided written informed consent and the study was approved by the local Medical Ethics Committee (Arnhem-Nijmegen; file no. 2002/188). The use of CSF leftovers from the control patients who had been seen as part of daily care in research projects was approved by the local Medical Ethics Committee.

### Mass Spectrometry—shotgun proteomics profiling

Total protein concentration in CSF was determined by using the 2D Quant kit (GE Healthcare Life Sciences, UK), according to the manufacturer’s protocol, and 400 µg total protein was used as input for profiling. All samples were loaded on an affinity removal column for the depletion of the 14 most abundant proteins (MARS-14, Agilent Technologies, Santa Clara, CA, USA). After tryptic digestion, CSF samples were fractionated in 20 fractions using high pH reversed-phase C18 LC and each fraction was subsequently analyzed by nanoflow liquid chromatography (Bruker Daltonics; nano-Advance) connected online to an ultra-high resolution quadrupole time-of-flight tandem mass spectrometer (Qq-TOF; Bruker Daltonics; maXis 4G ETD) as described previously^50^.

Raw MS data were analyzed by MaxQuant software version 1.5^51^ with pre-defined Qq-ToF parameter settings against the RefSeq (release 55) human protein sequence database. We set cysteine carbamidomethylation as a fixed modification, whereas N-terminal acetylation, methionine oxidation, and deamidation of glutamine and/or asparagine were set as variable modifications. For further statistical analysis, only peptides with intensity above the detection limit in at least 75% of the samples in one of the groups (PD, MSA, or non-neurological controls) were used.

### Mass spectrometry−targeted proteomics using SRM

For the selection of tryptic peptides for the SRM assay, additional criteria were used: (1) *p*-value below 0.05 determined by Mann−Whitney U test comparing PD vs. MSA; (2) ratio of intensity (PD:MSA) of at least 1.5; (3) intensity values above MS detection limit in at least 75% of samples in both PD and MSA groups; (4) peptide length of maximal 20 amino acids; (5) uniqueness (assignment to only one protein); (6) information available in Uniprot^52^ or PeptideAtlas^53^; (7) exclusion of peptides with susceptibility to post-translational or chemical modifications, such as methionine and cysteine oxidation, a potential deamidation site, or N-terminal cyclization.

The CSF samples for SRM and MS analysis were processed in randomized order using 50 µL of CSF from each patient as input. Prior to protein digestion, samples were subjected to overnight freeze-drying to concentrate the sample. On the next day, the sample was reconstituted with 4.3 µL of 8 M urea solution in 10 mM Tris-HCL, and diluted with 4.3 µL 10 mM Tris-HCL to reach a final urea concentration of 4 M. Protein reduction was performed by incubation with 0.5 µL of 10 mM dithiothreitol for 30 min at room temperature, followed by alkylation by incubation with 0.5 µL of 50 mM 2-chloroacetamide for 30 min at room temperature, kept in the dark. Samples were then incubated with 1 µL of 0.5 μg/μl Lysyl endopeptidase C for 3 h at room temperature, resulting in a volume of 10.6 µL, which was subsequently diluted four times with 31.6 µL of 50 mM ammonium bicarbonate and incubated with 1 µL of trypsin (1 μg trypsin/50 μg protein) for 4 h at 37 °C. Digestion reaction was stopped by adding 4.8 µL of 10% trifluoroacetic acid. A cocktail of synthesized isotope-labelled “heavy” peptides (JPT, Germany) on the C-termini of the target peptides at either a lysine (^13^C_6_^15^N_2_) or arginine (^13^C_6_^15^N_4_) residue was added to each sample to allow peptide identification and relative quantification. Samples were cleaned by passing them over a 0.22 µm filter and stored at −80 °C until MS analysis.

Samples (2 µL) were subjected to LC-MS analysis in randomized order on the Acquity MClass UPLC Xevo TQ-S (Waters), coupled with an ionKey/MS system using a Waters peptide BEH C18, 130 Å, 1.7 μm, 150 μm × 100 mm ionKey column for chromatographic separation using a 30 min linear gradient of acetonitrile ranging from 3 to 35% with 0.1% formic acid at a flow rate of 2 μl/min.

To optimize SRM settings in the SRM method development step, we used a pooled trypsin digested CSF sample spiked in with a cocktail of heavy labelled peptides (final concentration of 10 fmol for each peptide), and specifically, the cone voltage and collision energy were optimized for each peptide fragment. For each peptide, we started with a selection of at least 10 peptide fragments per precursor (transitions). For the final multiplex SRM assay, at least 2 transitions with the highest signal intensity and lack of interference were selected for each peptide target. For each peptide fragment, retention time windows of 1 min were used, allowing both endogenous and heavy labelled peptides to have at least 8 data points per chromatographic peak with an average dynamically dwell time of 250 ms.

Our newly developed SRM method was validated using a pooled digested CSF sample mixed with a cocktail of heavy labelled peptides and the following criteria were investigated: (1) linearity to provide a calibration curve, by using a dilution series of the cocktail of heavy labelled peptides (0, 0.625, 1.25, 2.5, 5, 10, 20, and 40 fmol) spiked into pooled digested CSF in three replicates. Peptide fragments with a linear regression coefficient (*R*^2^) below 0.7 were excluded. The calibration curve was used to determine the best heavy labelled peptide concentration for the clinical samples and a new peptide cocktail was prepared; (2) intra-assay variation < 20% for 1 pooled digested CSF sample injected five times on the same day; (3) inter-assay variation < 20% for 1 digested CSF sample measured on 10 different days; (4) inter-assay sample preparation < 20% for 5 identical aliquots of pooled CSF samples, all digested and measured on the same day; (5) sample stability on the autosampler which was set at 10 °C by injecting 1 sample repetitively from the same plate every 4 h for 24 h; (6) freeze/thaw effect < 20% for 1 pooled and digested CSF sample subjected to up to 5 freeze/thaw cycles; (7) freeze/thaw effect < 20% for a pooled CSF sample subjected to 3 freeze/thaw cycles prior to the digestion procedure. CV was calculated between technical replicates and a CV of 20% was regarded as acceptable. Results of these 7 criteria for the SRM method validation for all included tryptic peptides are shown in Supplementary Table 1a−1g respectively. To correct for possible variation between the days of sample preparation of the clinical cohort, two pooled CSF samples were included as quality controls in each digestion cycle (see Supplementary Table 1h).

Total protein concentration in CSF of the clinical cohort was determined by turbidimetric benzethonium chloride method using a Cobas 8000 instrument (Roche Diagnostics, Switzerland) for automated measurement.

### Data analysis

Skyline software version 20.1 (MacCoss Lab, University of Washington, USA) was used to process raw data from SRM assay to confirm peak detection, correct integration, and calculation of the peak area^54^. For data analysis, the relative quantification was determined by calculation of the ratio between endogenous and heavy labelled peptides. We normalized each ratio of endogenous : heavy labelled peptides for total CSF protein concentration as these markers were identified in the proteomics profiling where also a normalization on total protein content was applied.

Analyses were performed in R software version 3.5.3 (Austria), IBM SPSS Statistics 25 (Armonk, NY, USA), or GraphPad Prism 5 (La Jolla, CA, USA). Groups were compared by using two-sided Student’s T-test or Mann−Whitney U test in the case of two groups depending on the data distribution (parametric or non-parametric), and two-sided analysis of variance (ANOVA) with Bonferroni’s multiple correction as a post hoc test or Kruskal−Wallis one-way analysis of variance with Dunn’s as post hoc test when more than two groups were analyzed. Rank analysis of covariance was used for group comparisons taking age as a covariate, including Bonferroni’s multiple correction as a post hoc test. Correlation of peptides with age and clinical parameters, such as disease duration, UPDRS, ICARS, MMSE scores, was performed using Spearman’s rank correlation coefficient. Random forest was applied for multivariate analysis to generate decision trees to improve group discrimination. The models generated by random forest were developed in 70% of our cohort and validated in 30%. For random forest analysis, an imputation method (Amelia II, R package) was used to fill in missing values^55^. Receiver operating characteristic (ROC) was used to determine the diagnostic accuracy by calculating the area under the curve (AUC).

### Reporting summary

Further information on research design is available in the Nature Research Reporting Summary linked to this article.

## Supplementary information

Supplementary Information

Reporting Summary

## Data Availability

The mass spectrometry shotgun proteomics profiling data have been deposited to the ProteomeXchange Consortium via the PRIDE^56^ partner repository with the dataset identifier PXD028842, and the SRM data have been deposited via PanoramaWeb and can be accessed via https://panoramaweb.org/neurochemistry_PD.url with the ProteomeXchange ID PXD028888.

## Code availability

Data analysis was performed using R version 3.5.3. The packages used were: reprtree v0.6, plotrix v3.8-1, tree v1.0-39, Amelia v1.7.6, Rcpp v1.0.6, rowr v1.1.3, rpart.plot v3.0.9, data.table v1.14.0, caret v6.0-86, lattice v0.20-38, rpart v4.1-15, gridExtra v2.3, reshape2 v1.4.4, ggpubr v0.4.0, ggplot2 v3.3.3, plyr v1.8.6, pROC v1.17.0.1, randomForest v4.6-14, readxl v1.3.1. The script is freely accessible at https://github.com/CharlotteKaffa/biomarkers-for-parkinsonism.
