# Supplementary material for: Identification of cerebrospinal fluid biomarkers for parkinsonism using a proteomics approach
Source: NPJ Parkinsons Dis. 2021 Nov 30;7:107. doi: 10.1038/s41531-021-00249-9 (PMC8633286; doi:10.1038/s41531-021-00249-9)
Supplement: Supplementary file 1 — Supplementary Information [file 41531_2021_249_MOESM1_ESM.pdf]

Supplementary Table 1: Results of SRM assay validation
a) linearity of spiked, heavy labelled peptides

Peptide Sequence	Protein name	Fragment	mean R^2	Fragment function
GFYFSR	Insulin-like growth factor II	Y [y4]	0,997	quantifier
		F [y3]	0,991	qualifier
		Y [y4]	0,988	qualifier
VQLSEFSPPGSR	Protocadherin Fat 2	L [y10]	0,998	quantifier
		S [y9]	0,988	qualifier
DDDFTTWTQLAK	Protein O-linked-mannose beta-1,2-N-acetylglucosaminyltransferase 1	T [y8]	0,899	qualifier
		T [y7]	0,906	quantifier
		T [y5]	0,881	qualifier
ALYYDLISSPDIHGTYK	Pigment epithelium-derived factor	S [y10]	0,917	quantifier
		P [y8]	0,917	qualifier
		P [y8]	0,937	qualifier
HVLFGTVGVPEHTYR	Thy-1 membrane glycoprotein	G [y8]	0,989	qualifier
		P [y6]	0,993	quantifier
		P [y6]	0,990	qualifier
FLDTGVVQSDR	Multiple epidermal growth factor-like domains protein 8	D [y9]	0,993	quantifier
		T [y8]	0,974	qualifier
		L [b2]	0,989	qualifier
NVALVSGDTENAK	Extracellular matrix protein 1	A [y11]	0,994	qualifier
		V [y9]	0,900	qualifier
		S [y8]	0,946	quantifier
LALFPDK	Neuroblastoma suppressor of tumorigenicity 1	A [y6]	0,996	qualifier
		L [y5]	0,998	quantifier
		F [y4]	0,997	qualifier
VFNTPEGVPSAPSSLK	Neuronal cell adhesion molecule	P [y12]	0,995	quantifier
		F [b2]	0,997	qualifier
		T [b4]	0,994	qualifier
SFPLSSEHAK	Cadherin-2	L [y7]	0,950	qualifier
		S [y6]	0,989	qualifier
		P [y8]	0,974	quantifier

LTGISDPVTVK	Noelin	G [y9]	0,995	quantifier
		S [y7]	0,948	qualifier
FEAFEEDR	Seizure 6-like protein 2	A [y6]	0,996	quantifier
		F [y5]	0,991	qualifier
		A [b3]	0,987	qualifier
FLEQELETITIPDLR	Phospholipid transfer protein	T [y6]	0,798	qualifier
		I [y5]	0,797	qualifier
		P [y4]	0,797	quantifier
LSPYVNYQFR	Neurofascin	V [y6]	0,979	qualifier
		N [y5]	0,992	qualifier
		P [y8]	0,986	quantifier
VLEYLNQEK	Secretogranin-2	E [y7]	0,997	quantifier
		Y [y6]	0,997	qualifier
		N [y4]	0,996	qualifier
SYLEITPSR	Inter-alpha-trypsin inhibitor heavy chain H5	L [y7]	0,997	qualifier
		E [y6]	0,991	qualifier
		I [y5]	0,994	quantifier
YGFIEGHVVIPR	CD44 antigen	E [y8]	0,985	qualifier
		V [y5]	0,960	qualifier
		V [y4]	0,967	quantifier
VESLEQEAANER	Amyloid-beta precursor protein	E [y8]	0,996	qualifier
		Q [y7]	0,980	qualifier
		A [y5]	0,981	quantifier
NLLDLR	SLIT and NTRK-like protein 1	L [y4]	0,990	quantifier
		D [y3]	0,985	qualifier
		L [y2]	0,992	qualifier
LTVFPDGTLEVR	Leucine-rich repeat and immunoglobulin-like domain-containing nogo receptor-interacting protein 1	P [y8]	0,961	quantifier
		F [y9]	0,942	qualifier
		P [y8]	0,985	qualifier
AFQVWSDVTPLR	72 kDa type IV collagenase	W [y8]	0,959	quantifier
		S [y7]	0,955	qualifier
		T [y4]	0,966	qualifier

AVVEVDESGTR	Plasma serine protease inhibitor	E [y8]	0,993	quantifier
		V [y7]	0,987	qualifier
		D [y6]	0,990	qualifier
LQAPVWEFK	Ceroid-lipofuscinosis neuronal protein 5	A [y7]	0,991	quantifier
		P [y6]	0,981	qualifier
		F [y2]	0,954	qualifier
LFEELVR	Pyruvate kinase PKM	E [y5]	0,986	quantifier
		E [y4]	0,993	qualifier
		L [y3]	0,973	qualifier
SQETGDLDVGGLQETDK	Fibulin-1	G [y8]	0,994	quantifier
		Q [b2]	0,996	qualifier
		E [b3]	0,982	qualifier
GAAVSNNIVVRPSR	Neuronal cell adhesion molecule	A [y12]	0,981	quantifier
		N [y9]	0,982	qualifier
SFQTGLFTAAR	Vitamin K-dependent protein S	T [y8]	0,988	quantifier
		G [y7]	0,985	qualifier
		F [y5]	0,980	qualifier
VGIPENAPIGTLLLR	Protocadherin gamma-C5	P [y12]	0,924	qualifier
		P [y8]	0,933	qualifier
		P [y12]	0,927	quantifier
FDFNAFR	Mannosyl-oligosaccharide 1,2-alpha-mannosidase IC	D [y6]	0,988	qualifier
		F [y5]	0,993	quantifier
		N [y4]	0,994	qualifier
TFTLLDPK	N-acetylmuramoyl-L-alanine amidase	L [y5]	0,992	quantifier
		L [y4]	0,989	qualifier
		D [y3]	0,990	qualifier
TSDQIHFFFAK	Antithrombin-III	A [y2]	0,985	qualifier
		S [y10]	0,981	quantifier
		D [y9]	0,978	qualifier
TDGAAPNVAPSDVGGGGGR	Contactin-1	P [y14]	0,980	quantifier
		A [y11]	0,826	qualifier

Linear curve of heavy labeled peptides diluted from 0 to 40 fmol in a CSF digested pool, based on the ratio of endogenous and heavy labeled peptides.

Supplementary Table 1: Results of SRM assay validation
b) Intra-assay variation

Peptide Sequence	Protein name	mean	std dev	CV%
GFYFSR	Insulin-like growth factor II	2,05	0,03	1,55%
VQLSEFSPPGSR	Protocadherin Fat 2	0,07	0,01	7,77%
DDDFTTWTQLAK	Protein O-linked-mannose beta-1,2-N-acetylglucosaminyltransferase 1	0,16	0,03	17,05%
ALYYDLISSPDIHGTYK	Pigment epithelium-derived factor	60,94	6,85	11,24%
HVLFGTVGVPEHTYR	Thy-1 membrane glycoprotein	2,25	0,08	3,72%
FLDTGVVQSDR	Multiple epidermal growth factor-like domains protein 8	0,33	0,03	7,77%
NVALVSGDTENAK	Extracellular matrix protein 1	1,32	0,06	4,77%
LALFPDK	Neuroblastoma suppressor of tumorigenicity 1	3,89	0,24	6,18%
VFNTPEGVPSAPSSLK	Neuronal cell adhesion molecule	0,94	0,04	3,74%
SFPLSSEHAK	Cadherin-2	0,24	0,05	20,40%
LTGISDPVTVK	Noelin	0,03	0,00	7,96%
FEAFEEDR	Seizure 6-like protein 2	0,13	0,00	3,54%
FLEQELETITIPDLR	Phospholipid transfer protein	473,82	21,94	4,63%
LSPYVNYQFR	Neurofascin	0,17	0,01	5,25%
VLEYLNQEK	Secretogranin-2	2,51	0,03	1,15%
SYLEITPSR	Inter-alpha-trypsin inhibitor heavy chain H5	0,30	0,02	5,86%
YGFIEGHVVIPR	CD44 antigen	3,14	0,53	16,88%
VESLEQEAANER	Amyloid-beta precursor protein	8,04	0,18	2,21%
NLLDLR	SLIT and NTRK-like protein 1	0,13	0,01	4,43%
LTVFPDGTLEVR	Leucine-rich repeat and immunoglobulin-like domain-containing nogo receptor-interacting protein 1	0,59	0,04	6,96%
AFQVWSDVTPLR	72 kDa type IV collagenase	1,81	0,13	7,34%
AVVEVDESGTR	Plasma serine protease inhibitor	1,04	0,02	2,18%
LQAPVWEFK	Ceroid-lipofuscinosis neuronal protein 5	0,16	0,01	3,53%
LFEELVR	Pyruvate kinase PKM	1,38	0,12	8,68%
SQETGDLDVGGLQETDK	Fibulin-1	55,11	2,38	4,31%
GAAVSNNIVVRPSR	Neuronal cell adhesion molecule	31,63	3,06	9,67%
SFQTGLFTAAR	Vitamin K-dependent protein S	1,77	0,11	6,21%
VGIPENAPIGTLLLR	Protocadherin gamma-C5	0,17	0,01	5,12%
FDFNAFR	Mannosyl-oligosaccharide 1,2-alpha-mannosidase IC	0,13	0,01	7,02%

TFTLLDPK	N-acetylmuramoyl-L-alanine amidase	2,48	0,30	11,96%
TSDQIHFFFAK	Antithrombin-III	28,23	1,43	5,08%
TDGAAPNVAPSDVGGGGGR	Contactin-1	2,33	0,08	3,50%

Intra-assay variation of one digested pooled CSF injected five times on the same day. Mean was calculated based on ratio of endogenous : heavy labeled peptides, multiplied by 10 for a better visualization. Standard deviation (std dev) was calculated based on replicates. Coefficient of variation (CV) was calculated by dividing the standard deviation by the mean.

Supplementary Table 1: Results of SRM assay validation
c) Inter-assay variation

Peptide Sequence	Protein name	mean	std dev	CV%
GFYFSR	Insulin-like growth factor II	5,43	0,12	2,15%
VQLSEFSPPGSR	Protocadherin Fat 2	0,12	0,01	8,61%
DDDFTTWTQLAK	Protein O-linked-mannose beta-1,2-N-acetylglucosaminyltransferase 1	0,34	0,02	7,10%
ALYYDLISSPDIHGTYK	Pigment epithelium-derived factor	14,00	0,99	7,11%
HVLFGTVGVPEHTYR	Thy-1 membrane glycoprotein	3,52	0,06	1,75%
FLDTGVVQSDR	Multiple epidermal growth factor-like domains protein 8	2,68	0,12	4,45%
NVALVSGDTENAK	Extracellular matrix protein 1	4,64	0,06	1,35%
LALFPDK	Neuroblastoma suppressor of tumorigenicity 1	9,53	0,25	2,62%
VFNTPEGVPSAPSSLK	Neuronal cell adhesion molecule	4,98	0,12	2,32%
SFPLSSEHAK	Cadherin-2	3,34	0,21	6,44%
LTGISDPVTVK	Noelin	0,23	0,02	8,97%
FEAFEEDR	Seizure 6-like protein 2	1,33	0,01	1,06%
FLEQELETITIPDLR	Phospholipid transfer protein	1,93	0,01	0,52%
LSPYVNYQFR	Neurofascin	1,20	0,06	4,77%
VLEYLNQEK	Secretogranin-2	5,45	0,09	1,61%
SYLEITPSR	Inter-alpha-trypsin inhibitor heavy chain H5	3,07	0,19	6,22%
YGFIEGHVVIPR	CD44 antigen	2,86	0,04	1,42%
VESLEQEAANER	Amyloid-beta precursor protein	5,27	0,07	1,39%
NLLDLR	SLIT and NTRK-like protein 1	1,25	0,11	8,73%
LTVFPDGTLEVR	Leucine-rich repeat and immunoglobulin-like domain-containing nogo receptor-interacting protein 1	1,56	0,06	3,69%
AFQVWSDVTPLR	72 kDa type IV collagenase	2,03	0,03	1,53%
AVVEVDESGTR	Plasma serine protease inhibitor	5,80	0,18	3,04%
LQAPVWEFK	Ceroid-lipofuscinosis neuronal protein 5	1,11	0,04	3,22%
LFEELVR	Pyruvate kinase PKM	5,78	0,14	2,41%
SQETGDLDVGGLQETDK	Fibulin-1	13,87	0,25	1,77%
GAAVSNNIVVRPSR	Neuronal cell adhesion molecule	9,28	0,56	6,00%
SFQTGLFTAAR	Vitamin K-dependent protein S	3,45	0,09	2,75%
VGIPENAPIGTLLLR	Protocadherin gamma-C5	0,38	0,03	7,33%
FDFNAFR	Mannosyl-oligosaccharide 1,2-alpha-mannosidase IC	0,90	0,01	1,44%

TFTLLDPK	N-acetylmuramoyl-L-alanine amidase	4,82	0,16	3,33%
TSDQIHFFFAK	Antithrombin-III	6,14	0,19	3,11%
TDGAAPNVAPSDVGGGGGR	Contactin-1	5,22	0,18	3,48%

Inter-assay variation of one digested pooled CSF injected on ten different days. Mean was calculated based on ratio of endogenous : heavy labeled peptides, multiplied by 10 for a better visualization. Standard deviation (std dev) was calculated based on replicates. Coefficient of variation (CV) was calculated by dividing the standard deviation by the mean.

Supplementary Table 1: Results of SRM assay validation
d) Inter-assay variation in sample preparation

Peptide Sequence	Protein name	mean	std dev	CV%
GFYFSR	Insulin-like growth factor II	1,57	0,15	9,33%
VQLSEFSPPGSR	Protocadherin Fat 2	0,05	0,00	10,05%
DDDFTTWTQLAK	Protein O-linked-mannose beta-1,2-N-acetylglucosaminyltransferase 1	0,10	0,01	14,48%
ALYYDLISSPDIHGTYK	Pigment epithelium-derived factor	43,53	2,48	5,70%
HVLFGTVGVPEHTYR	Thy-1 membrane glycoprotein	1,41	0,10	6,92%
FLDTGVVQSDR	Multiple epidermal growth factor-like domains protein 8	0,23	0,02	11,07%
NVALVSGDTENAK	Extracellular matrix protein 1	0,90	0,07	7,42%
LALFPDK	Neuroblastoma suppressor of tumorigenicity 1	3,03	0,10	3,19%
VFNTPEGVPSAPSSLK	Neuronal cell adhesion molecule	0,49	0,02	4,69%
SFPLSSEHAK	Cadherin-2	1,65	0,21	12,92%
LTGISDPVTVK	Noelin	0,02	0,00	8,44%
FEAFEEDR	Seizure 6-like protein 2	0,09	0,01	6,40%
FLEQELETITIPDLR	Phospholipid transfer protein	116,46	12,31	10,57%
LSPYVNYQFR	Neurofascin	0,10	0,00	3,59%
VLEYLNQEK	Secretogranin-2	1,81	0,07	3,77%
SYLEITPSR	Inter-alpha-trypsin inhibitor heavy chain H5	0,20	0,02	9,01%
YGFIEGHVVIPR	CD44 antigen	2,12	0,06	3,01%
VESLEQEAANER	Amyloid-beta precursor protein	6,42	0,22	3,38%
NLLDLR	SLIT and NTRK-like protein 1	0,10	0,01	7,38%
LTVFPDGTLEVR	Leucine-rich repeat and immunoglobulin-like domain-containing nogo receptor-interacting protein 1	0,38	0,03	7,36%
AFQVWSDVTPLR	72 kDa type IV collagenase	1,10	0,06	5,67%
AVVEVDESGTR	Plasma serine protease inhibitor	0,42	0,02	3,59%
LQAPVWEFK	Ceroid-lipofuscinosis neuronal protein 5	0,11	0,01	7,03%
LFEELVR	Pyruvate kinase PKM	0,93	0,05	4,91%
SQETGDLDVGGLQETDK	Fibulin-1	38,08	2,09	5,50%
GAAVSNNIVVRPSR	Neuronal cell adhesion molecule	20,35	1,49	7,33%
SFQTGLFTAAR	Vitamin K-dependent protein S	1,31	0,16	12,24%
VGIPENAPIGTLLLR	Protocadherin gamma-C5	0,10	0,01	10,27%
FDFNAFR	Mannosyl-oligosaccharide 1,2-alpha-mannosidase IC	0,10	0,00	1,89%

TFTLLDPK	N-acetylmuramoyl-L-alanine amidase	1,69	0,06	3,70%
TSDQIHFFFAK	Antithrombin-III	18,21	1,18	6,49%
TDGAAPNVAPSDVGGGGGR	Contactin-1	1,13	0,09	7,95%

Inter-assay variation of sample preparation (by digesting aliquots of five identical pooled CSF samples on the same day and measure them all on the same day). Mean was calculated based on ratio of endogenous : heavy labeled peptides, multiplied by 10 for a better visualization. Standard deviation (std dev) was calculated based on replicates. Coefficient of variation (CV) was calculated by dividing the standard deviation by the mean.

Supplementary Table 1: Results of SRM assay validation
e) Sample stability during 24h on the autosampler

Peptide Sequence	Protein name	mean	std dev	CV%
GFYFSR	Insulin-like growth factor II	1,99	0,11	5,48%
VQLSEFSPPGSR	Protocadherin Fat 2	0,07	0,01	7,66%
DDDFTTWTQLAK	Protein O-linked-mannose beta-1,2-N-acetylglucosaminyltransferase 1	0,17	0,02	13,15%
ALYYDLISSPDIHGTYK	Pigment epithelium-derived factor	67,44	8,77	13,01%
HVLFGTVGVPEHTYR	Thy-1 membrane glycoprotein	2,25	0,08	3,63%
FLDTGVVQSDR	Multiple epidermal growth factor-like domains protein 8	0,33	0,02	6,65%
NVALVSGDTENAK	Extracellular matrix protein 1	1,34	0,24	17,59%
LALFPDK	Neuroblastoma suppressor of tumorigenicity 1	3,89	0,17	4,45%
VFNTPEGVPSAPSSLK	Neuronal cell adhesion molecule	0,92	0,03	3,49%
SFPLSSEHAK	Cadherin-2	0,19	0,05	26,10%
LTGISDPVTVK	Noelin	0,03	0,01	16,52%
FEAFEEDR	Seizure 6-like protein 2	0,14	0,01	7,83%
FLEQELETITIPDLR	Phospholipid transfer protein	480,58	24,36	5,07%
LSPYVNYQFR	Neurofascin	0,17	0,01	7,88%
VLEYLNQEK	Secretogranin-2	2,53	0,11	4,27%
SYLEITPSR	Inter-alpha-trypsin inhibitor heavy chain H5	0,31	0,02	6,87%
YGFIEGHVVIPR	CD44 antigen	3,05	0,46	14,96%
VESLEQEAANER	Amyloid-beta precursor protein	8,21	0,87	10,62%
NLLDLR	SLIT and NTRK-like protein 1	0,13	0,01	4,62%
LTVFPDGTLEVR	Leucine-rich repeat and immunoglobulin-like domain-containing nogo receptor-interacting protein 1	0,58	0,05	8,79%
AFQVWSDVTPLR	72 kDa type IV collagenase	1,77	0,10	5,92%
AVVEVDESGTR	Plasma serine protease inhibitor	1,05	0,06	5,68%
LQAPVWEFK	Ceroid-lipofuscinosis neuronal protein 5	0,15	0,01	4,47%
LFEELVR	Pyruvate kinase PKM	1,35	0,09	6,92%
SQETGDLDVGGLQETDK	Fibulin-1	55,98	5,33	9,53%
GAAVSNNIVVRPSR	Neuronal cell adhesion molecule	31,41	4,40	14,02%
SFQTGLFTAAR	Vitamin K-dependent protein S	1,74	0,12	7,05%
VGIPENAPIGTLLLR	Protocadherin gamma-C5	0,17	0,01	7,12%
FDFNAFR	Mannosyl-oligosaccharide 1,2-alpha-mannosidase IC	0,13	0,01	6,02%

TFTLLDPK	N-acetylmuramoyl-L-alanine amidase	2,39	0,25	10,62%
TSDQIHFFFAK	Antithrombin-III	28,49	1,36	4,76%
TDGAAPNVAPSDVGGGGGR	Contactin-1	2,22	0,21	9,52%

Sample stability on the plate by injecting one digested pooled CSF every 4 h for 24 h. Mean was calculated based on ratio of endogenous : heavy labeled peptides, multiplied by 10 for a better visualization. Standard deviation (std dev) was calculated based on replicates. Coefficient of variation (CV) was calculated by dividing the standard deviation by the mean.

Supplementary Table 1: Results of SRM assay validation
f) Stability (freeze / thaw effect) for digested samples

Peptide Sequence	Protein name	mean	std dev	CV%
GFYFSR	Insulin-like growth factor II	1,58	0,03	1,92%
VQLSEFSPPGSR	Protocadherin Fat 2	0,05	0,00	7,09%
DDDFTTWTQLAK	Protein O-linked-mannose beta-1,2-N-acetylglucosaminyltransferase 1	0,11	0,01	12,57%
ALYYDLISSPDIHGTYK	Pigment epithelium-derived factor	49,06	6,28	12,80%
HVLFGTVGVPEHTYR	Thy-1 membrane glycoprotein	1,42	0,02	1,19%
FLDTGVVQSDR	Multiple epidermal growth factor-like domains protein 8	0,23	0,01	5,95%
NVALVSGDTENAK	Extracellular matrix protein 1	0,92	0,07	7,39%
LALFPDK	Neuroblastoma suppressor of tumorigenicity 1	3,11	0,09	2,78%
VFNTPEGVPSAPSSLK	Neuronal cell adhesion molecule	0,52	0,03	5,17%
SFPLSSEHAK	Cadherin-2	0,14	0,02	15,72%
LTGISDPVTVK	Noelin	0,02	0,00	7,51%
FEAFEEDR	Seizure 6-like protein 2	0,09	0,00	1,70%
FLEQELETITIPDLR	Phospholipid transfer protein	116,08	4,69	4,04%
LSPYVNYQFR	Neurofascin	0,10	0,01	5,64%
VLEYLNQEK	Secretogranin-2	1,78	0,05	2,86%
SYLEITPSR	Inter-alpha-trypsin inhibitor heavy chain H5	0,21	0,01	6,60%
YGFIEGHVVIPR	CD44 antigen	2,10	0,06	2,99%
VESLEQEAANER	Amyloid-beta precursor protein	6,38	0,18	2,82%
NLLDLR	SLIT and NTRK-like protein 1	0,10	0,01	8,12%
LTVFPDGTLEVR	Leucine-rich repeat and immunoglobulin-like domain-containing nogo receptor-interacting protein 1	0,38	0,02	5,32%
AFQVWSDVTPLR	72 kDa type IV collagenase	1,06	0,02	1,49%
AVVEVDESGTR	Plasma serine protease inhibitor	0,42	0,02	4,08%
LQAPVWEFK	Ceroid-lipofuscinosis neuronal protein 5	0,11	0,01	5,86%
LFEELVR	Pyruvate kinase PKM	0,95	0,03	3,20%
SQETGDLDVGGLQETDK	Fibulin-1	40,19	1,42	3,52%
GAAVSNNIVVRPSR	Neuronal cell adhesion molecule	22,64	2,21	9,77%
SFQTGLFTAAR	Vitamin K-dependent protein S	1,28	0,05	4,22%
VGIPENAPIGTLLLR	Protocadherin gamma-C5	0,09	0,01	7,63%
FDFNAFR	Mannosyl-oligosaccharide 1,2-alpha-mannosidase IC	0,09	0,00	4,62%

TFTLLDPK	N-acetylmuramoyl-L-alanine amidase	1,73	0,06	3,62%
TSDQIHFFFAK	Antithrombin-III	18,45	0,37	2,03%
TDGAAPNVAPSDVGGGGGR	Contactin-1	1,12	0,04	3,58%

Freeze / thaw effect using one digested pooled CSF, subjected to 5 freeze / thaw cycles. Mean was calculated based on ratio of endogenous : heavy labeled peptides, multiplied by 10 for a better visualization. Standard deviation (std dev) was calculated based on replicates. Coefficient of variation (CV) was calculated by dividing the standard deviation by the mean.

Supplementary Table 1: Results of SRM assay validation

g) Stability of CSF samples subjected to freeze / thaw cycles prior to digestion

Peptide Sequence	Protein name	mean	std. dev.	CV%
GFYFSR	Insulin-like growth factor II	2,02	0,11	5,32%
VQLSEFSPPGSR	Protocadherin Fat 2	0,08	0,01	13,15%
DDDFTTWTQLAK	Protein O-linked-mannose beta-1,2-N-acetylglucosaminyltransferase 1	0,15	0,01	8,13%
ALYYDLISSPDIHGTYK	Pigment epithelium-derived factor	104,42	13,83	13,25%
HVLFGTVGVPEHTYR	Thy-1 membrane glycoprotein	2,43	0,38	15,68%
FLDTGVVQSDR	Multiple epidermal growth factor-like domains protein 8	0,33	0,03	9,40%
NVALVSGDTENAK	Extracellular matrix protein 1	1,51	0,23	14,94%
LALFPDK	Neuroblastoma suppressor of tumorigenicity 1	4,54	0,37	8,11%
VFNTPEGVPSAPSSLK	Neuronal cell adhesion molecule	0,92	0,16	17,69%
SFPLSSEHAK	Cadherin-2	0,20	0,02	8,59%
LTGISDPVTVK	Noelin	0,03	0,01	23,27%
FEAFEEDR	Seizure 6-like protein 2	0,12	0,01	12,38%
FLEQELETITIPDLR	Phospholipid transfer protein	169,70	15,82	9,32%
LSPYVNYQFR	Neurofascin	0,19	0,01	6,27%
VLEYLNQEK	Secretogranin-2	2,53	0,37	14,58%
SYLEITPSR	Inter-alpha-trypsin inhibitor heavy chain H5	0,34	0,02	6,54%
YGFIEGHVVIPR	CD44 antigen	3,38	0,58	17,05%
VESLEQEAANER	Amyloid-beta precursor protein	9,82	1,24	12,61%
NLLDLR	SLIT and NTRK-like protein 1	0,17	0,03	15,09%
LTVFPDGTLEVR	Leucine-rich repeat and immunoglobulin-like domain-containing nogo receptor-interacting protein 1	0,61	0,11	17,57%
AFQVWSDVTPLR	72 kDa type IV collagenase	1,77	0,26	14,93%
AVVEVDESGTR	Plasma serine protease inhibitor	1,17	0,11	9,09%
LQAPVWEFK	Ceroid-lipofuscinosis neuronal protein 5	0,19	0,03	13,65%
LFEELVR	Pyruvate kinase PKM	1,68	0,19	11,52%
SQETGDLDVGGLQETDK	Fibulin-1	59,12	8,27	13,99%
GAAVSNNIVVRPSR	Neuronal cell adhesion molecule	35,76	2,13	5,96%
SFQTGLFTAAR	Vitamin K-dependent protein S	1,85	0,20	10,68%
VGIPENAPIGTLLLR	Protocadherin gamma-C5	0,15	0,02	14,69%
FDFNAFR	Mannosyl-oligosaccharide 1,2-alpha-mannosidase IC	0,16	0,02	14,68%

TFTLLDPK	N-acetylmuramoyl-L-alanine amidase	2,58	0,26	10,10%
TSDQIHFFFAK	Antithrombin-III	32,37	3,66	11,30%
TDGAAPNVAPSDVGGGGGR	Contactin-1	2,30	0,08	3,54%

Freeze / thaw effect using one pooled CSF sample subjected to 3 freeze / thaw cycles prior to the digestion steps. Mean was calculated based on ratio of endogenous : heavy labeled peptides, multiplied by 10 for a better visualization. Standard deviation (std dev) was calculated based on replicates. Coefficient of variation (CV) was calculated by dividing the standard deviation by the mean.

Supplementary Table 1: Results of SRM assay validation

h) Variation in SRM measures of quality control (QC) samples

Peptide sequence	Protein name	Ratio mean	std dev	CV%
GFYFSR	Insulin-like growth factor II	5,79	0,69	11,92%
VQLSEFSPPGSR	Protocadherin Fat 2	0,12	0,01	9,00%
DDDFTTWTQLAK	Protein O-linked-mannose beta-1,2-N-acetylglucosaminyltransferase 1	0,44	0,07	16,30%
ALYYDLISSPDIHGTYK	Pigment epithelium-derived factor	20,20	1,07	5,30%
HVLFGTVGVPEHTYR	Thy-1 membrane glycoprotein	5,13	0,90	17,48%
FLDTGVVQSDR	Multiple epidermal growth factor-like domains protein 8	3,14	0,22	6,97%
NVALVSGDTENAK	Extracellular matrix protein 1	4,49	0,19	4,16%
LALFPDK	Neuroblastoma suppressor of tumorigenicity 1	10,13	0,30	2,92%
VFNTPEGVPSAPSSLK	Neuronal cell adhesion molecule	5,94	0,26	4,33%
SFPLSSEHAK	Cadherin-2	3,60	0,23	6,46%
LTGISDPVTVK	Noelin	0,34	0,02	4,70%
FEAFEEDR	Seizure 6-like protein 2	1,51	0,14	9,16%
FLEQELETITIPDLR	Phospholipid transfer protein	2,39	0,30	12,41%
LSPYVNYQFR	Neurofascin	1,56	0,12	7,76%
VLEYLNQEK	Secretogranin-2	4,48	0,18	3,99%
SYLEITPSR	Inter-alpha-trypsin inhibitor heavy chain H5	3,78	0,17	4,37%
YGFIEGHVVIPR	CD44 antigen	3,87	0,31	8,00%
VESLEQEAANER	Amyloid-beta precursor protein	5,28	0,44	8,28%
NLLDLR	SLIT and NTRK-like protein 1	1,49	0,15	10,05%
LTVFPDGTLEVR	Leucine-rich repeat and immunoglobulin-like domain-containing nogo receptor-interacting protein 1	2,23	0,16	7,35%
AFQVWSDVTPLR	72 kDa type IV collagenase	3,14	0,07	2,23%
AVVEVDESGTR	Plasma serine protease inhibitor	6,87	0,68	9,93%
LQAPVWEFK	Ceroid-lipofuscinosis neuronal protein 5	1,53	0,08	4,92%
LFEELVR	Pyruvate kinase PKM	6,47	0,39	6,09%
SQETGDLDVGGLQETDK	Fibulin-1	16,22	0,86	5,30%
GAAVSNNIVVRPSR	Neuronal cell adhesion molecule	9,97	1,12	11,21%
SFQTGLFTAAR	Vitamin K-dependent protein S	3,88	0,28	7,22%
VGIPENAPIGTLLLR	Protocadherin gamma-C5	0,57	0,04	7,68%
FDFNAFR	Mannosyl-oligosaccharide 1,2-alpha-mannosidase IC	0,96	0,06	5,93%

TFTLLDPK	N-acetylmuramoyl-L-alanine amidase	5,02	0,16	3,18%
TSDQIHFFFAK	Antithrombin-III	8,33	0,40	4,85%
TDGAAPNVAPSDVGGGGGR	Contactin-1	5,37	0,34	6,25%

Two digested pooled CSF samples were included as quality controls in each digestion cycle of the clinical cohort. Mean was calculated based on ratio of endogenous : heavy labeled peptides, multiplied by 10 for a better visualization. Standard deviation (std dev) was calculated based on replicates. Coefficient of variation (CV) was calculated by dividing the standard deviation by the mean.

Supplementary Table 2: Biochemical and clinical parameters included in models

	PD	MSA	PSP
age	n = 46<br>57.5 ± 10.0	n = 17<br>61.6 ± 7.9	n = 8<br>67.0 ± 6.6
NfL pg/mL	n = 46<br>1221 ± 660.1	n = 16<br>5298.5 ± 4579.0	n = 8<br>4461.3 ± 3889.0
phosphorylated tau pg/mL	n = 46<br>47.7 ± 16.2	n = 17<br>45.3 ± 12.9	n = 8<br>53.8 ± 15.7
ICARS	n = 42<br>2.8 ± 3.2	n = 13<br>9.5 ± 11.1	n = 6<br>10.7 ± 7.4
dysarthria cerebellar (yes / no)	n = 46<br>0 / 46	n = 17<br>5 / 12	n = 8<br>1 / 7
disease stage (normal / disturbed gait (walking independently / use of walking aids: intermittend / or permanent; / use of wheelchair: intermittend / or permanent))	n = 44<br>7 / 35 / 1 / 0 / 1 / 0	n = 17<br>0 / 13 / 2 / 1 / 1 / 0	n = 8<br>0 / 4 / 2 / 1 / 1 / 0
orthostatic pulse direct	n = 39<br>79.5 ± 20.2	n = 14<br>78.1 ± 17.3	n = 6<br>80.3 ± 9.2
UPDRS leg agility right leg (normal / slight / mild / moderate / severe)	n = 45<br>10 / 15 / 15 / 5 / 0	n = 17<br>2 / 8 / 6 / 1 / 0	n = 8<br>2 / 0 / 2 / 4 / 0
UPDRS postural stability (normal / slight / mild / moderate / severe)	n = 45<br>28 / 14 / 3 / 0 / 0	n = 17<br>1 / 5 / 9 / 2 / 0	n = 8<br>0 / 3 / 3 / 2 / 0
UPDRS bradykinesia (normal / slight / mild / moderate / severe)	n = 45<br>3 / 17 / 16 / 9 / 0	n = 17<br>1 / 3 / 8 / 5 / 0	n = 8<br>0 / 1 / 5 / 1 / 1
UPDRS right arm rigidity (normal / slight / mild / moderate / severe)	n = 45<br>6 / 14 / 19 / 6 / 0	n = 17<br>3 / 8 / 6 / 0 / 0	n = 8<br>2 / 3 / 2 / 0 / 1
Hoehn & Yahr stage (0 / 1 / 1.5 / 2 / 2.5 / 3 / 4 / 5)	n = 45<br>0 / 5 / 10 / 15 / 9 / 5 / 1 / 0	n = 17<br>1 / 1 / 1 / 0 / 7 / 5 / 2 / 0	n = 8<br>0 / 0 / 0 / 0 / 3 / 2 / 3 / 0
verbal fluency score	n = 42<br>10.2 ± 5.7	n = 15<br>9.7 ± 4.9	n = 8<br>4.5 ± 1.9
tandem gait score (normal / 1 side step / 2 or more side steps / unable to perform 4 consecutive steps)	n = 45<br>42 / 1 / 2 / 0	n = 16<br>3 / 2 / 8 / 3	n = 7<br>1 / 0 / 3 / 3
orthostatic hypotension 5 min	n = 39<br>132.1 ± 27.6	n = 15<br>115.3 ± 19.7	n = 6<br>141.8 ± 34.5

Values are expressed as mean ± standard deviation, or as number of patients. Abbreviations used: n: number of samples; PD: Parkinson's Disease; MSA: Multiple system atrophy; PSP: Progressive supranuclear palsy; UPDRS: Unified Parkinson’s Disease Rating Scale; ICARS: International Cooperative Ataxia Rating Scale; NfL: neurofilament light chain

Supplementary Methods

Quantification of established protein biomarkers in CSF

Previously published data on CSF analysis of neurofilament light chain (NfL), α-synuclein (α-syn), total tau, phosphorylated tau, amyloid-β42, and α-syn real-time quaking induced conversion (RT-QuIC) was used [1-5]. These established protein biomarkers were previously quantified as follows.

Commercially available ELISAs were used for the quantification of NfL (Uman Diagnostics, Umeå, Sweden), total tau (Innotest®, Fujirebio, Ghent, Belgium), phosphorylated tau $_{(181P)}$ (Innotest®, Fujirebio), and amyloid-β42 (Innotest®, Fujirebio).

For the quantification of α-syn, a previously developed ELISA was used [6], which was based on a previously described procedure [7] with several important modifications. In brief, a disposable flat-bottom microtiterplate (Nunc Maxisorp F96, Roskilde, Denmark) was coated with 100 µl antibody 211 (anti-human α-syn antibody, clone 211, Santa Cruz Biotechnology, CA, USA; 0.2 µg/ml in 0.20 M carbonate buffer pH 9.6) overnight at 4 °C. A plate washer (BioTek, Beun de Ronde, Abcoude, The Netherlands) was used to wash the plate five times with 250 µl PBS containing 0.05% Tween-20 (PBS washing buffer). All further incubations were performed at 37 °C, unless stated otherwise, and all measurements were performed in duplicate. Two hundred and fifty microliters of blocking buffer (2.5% gelatin in PBS washing buffer) were added and incubated for 2 h and the plate was subsequently washed five times with PBS washing buffer. Next, 100 µl alpha-synuclein solution (human α-syn recombinant protein; rPeptide, Bogart, GA, USA; from 0 to 500 ng/ml diluted in PBS) or CSF was added to each well and incubated for 2.5 h (in duplicate). Then, the plate was washed five times with PBS washing buffer and 100 µl of antibody FL-140 (rabbit anti-human α-syn antibody, clone FL-140, Santa Cruz Biotechnology), diluted 1:1000 in blocking buffer, was added for 1.5 h. Again, the plate was washed five times and 100 µl of horseradish peroxidase labeled goat anti-rabbit antibody (Jackson Immuno Research Laboratories, Baltimore, MD, USA; dilution 1:5000 in blocking buffer) was added and incubated for 1 h. After a final washing step 100 µl of a freshly prepared solution of tetramethyl benzidine (TMB) was applied and incubated for 15 min in the dark at room temperature. The reaction was stopped by addition of 50 µl 2N H2SO4 and the absorbance was measured at 450 nm in an ELISA plate reader (Tecan Sunrise, Salzburg, Austria).

The α-syn RT-QuIC assay was performed as previously described [8]. In brief, the RT-QuIC reaction buffer (RB) was composed of 100 mmol/L phosphate buffer (pH 8.2), 10 µmol/L thioflavin T (ThT), and 0.1 mg/mL human recombinant full-length (1–140 aa) alpha-synuclein (Stratech, Cambridge, UK). Each well of a black 96-well plate with a clear bottom (Nalgene Nunc International, Fisher Scientific Ltd, Loughborough, UK) contained 98 µL, 90 µL, or 85 µL RB (depending on the volume of seed added) and 37 ± 3 mg of 0.5 mm zirconium/silica beads (Thistle Scientific Ltd, Glasgow, UK). Reactions were seeded with 2 µL of working strength brain homogenate, 5 µL, 10 µL or 15 µL, of undiluted CSF to a final reaction volume of 100 µL. The plates were sealed with a plate sealer film (Fisher Scientific Ltd) and incubated in a BMG OPTIMA FluoSTAR plate reader at 30 °C for 120 h with intermittent shaking cycles: double orbital with 1 min shake (200 rpm), 14 min rest. ThT fluorescence measurements (450 nm excitation and 480 nm emission) were taken every 15 min.

Each sample was run in duplicate, allowing two negative control samples (reactions seeded with Sudden Death and Alzheimer's disease brain homogenate), one positive control (reaction seeded with Lewy body dementia brain homogenate), an unseeded reaction, and the CSF samples to be tested on one plate. A positive response was defined as a relative fluorescence unit value of >2 standard deviations above the mean of the negative controls at 120 hours in both of the CSF duplicates. If only 1 of 2 CSF samples was positive, the analysis was repeated in quadruplicate. A positive signal in 2 or more of the replicates was considered positive. Results were considered equivocal either because of a long lag phase (>80 hours vs ± 60 hours in the truly positive samples) or if 1 in 4 wells reacted.

References

1 Aerts, M. B., Esselink, R. A., Abdo, W. F., Bloem, B. R. & Verbeek, M. M. CSF alpha-synuclein does not differentiate between parkinsonian disorders. *Neurobiol Aging* **33**, 430 e431-433 (2012).

2 Herbert, M. K. et al. CSF levels of DJ-1 and tau distinguish MSA patients from PD patients and controls. *Parkinsonism Relat Disord* **20**, 112-115 (2014).

3 Aerts, M. B. et al. Ancillary investigations to diagnose parkinsonism: a prospective clinical study. *J Neurol* **262**, 346-356 (2015).

4 Herbert, M. K. et al. CSF Neurofilament Light Chain but not FLT3 Ligand Discriminates Parkinsonian Disorders. *Front Neurol* **6**, 91 (2015).

5 van Rumund, A. et al. alpha-Synuclein real-time quaking-induced conversion in the cerebrospinal fluid of uncertain cases of parkinsonism. *Ann Neurol* **85**, 777-781 (2019).

6 van Geel, W. J. et al. A more efficient enzyme-linked immunosorbent assay for measurement of alpha-synuclein in cerebrospinal fluid. *J Neurosci Methods* **168**, 182-185 (2008).

7 Tokuda, T. et al. Decreased alpha-synuclein in cerebrospinal fluid of aged individuals and subjects with Parkinson's disease. *Biochem Biophys Res Commun* **349**, 162-166 (2006).

8 Fairfoul, G. et al. Alpha-synuclein RT-QuIC in the CSF of patients with alpha-synucleinopathies. *Ann Clin Transl Neurol* **3**, 812-818 (2016).
