# Supplementary material for: Identification of cerebrospinal fluid biomarkers for parkinsonism using a proteomics approach
Source: NPJ Parkinsons Dis. 2021 Nov 30;7:107. doi: 10.1038/s41531-021-00249-9 (PMC8633286; doi:10.1038/s41531-021-00249-9)
Supplement: Supplementary file 2 — Reporting Summary [file 41531_2021_249_MOESM2_ESM.pdf]

nature portfolio

Corresponding author(s): M.M. Verbeek

Last updated by author(s): Oct 5, 2021

Reporting Summary

Nature Portfolio wishes to improve the reproducibility of the work that we publish. This form provides structure for consistency and transparency in reporting. For further information on Nature Portfolio policies, see our Editorial Policies and the Editorial Policy Checklist.

Statistics

For all statistical analyses, confirm that the following items are present in the figure legend, table legend, main text, or Methods section.

n/a	Confirmed	
☐	☒	The exact sample size (*n*) for each experimental group/condition, given as a discrete number and unit of measurement
☐	☒	A statement on whether measurements were taken from distinct samples or whether the same sample was measured repeatedly
☐	☒	The statistical test(s) used AND whether they are one- or two-sided<br>*Only common tests should be described solely by name; describe more complex techniques in the Methods section.*
☐	☒	A description of all covariates tested
☐	☒	A description of any assumptions or corrections, such as tests of normality and adjustment for multiple comparisons
☐	☒	A full description of the statistical parameters including central tendency (e.g. means) or other basic estimates (e.g. regression coefficient) AND variation (e.g. standard deviation) or associated estimates of uncertainty (e.g. confidence intervals)
☐	☒	For null hypothesis testing, the test statistic (e.g. *F*, *t*, *r*) with confidence intervals, effect sizes, degrees of freedom and *P* value noted<br>*Give P values as exact values whenever suitable.*
☒	☐	For Bayesian analysis, information on the choice of priors and Markov chain Monte Carlo settings
☒	☐	For hierarchical and complex designs, identification of the appropriate level for tests and full reporting of outcomes
☒	☐	Estimates of effect sizes (e.g. Cohen's *d*, Pearson's *r*), indicating how they were calculated

Our web collection on statistics for biologists contains articles on many of the points above.

Software and code

Policy information about availability of computer code

Data collection	MaxQuant software version 1.5; Skyline software version 20.1
Data analysis	R software version 3.5.3; IBM SPSS Statistics version 25.0; GraphPad Prism, version 5.03

For manuscripts utilizing custom algorithms or software that are central to the research but not yet described in published literature, software must be made available to editors and reviewers. We strongly encourage code deposition in a community repository (e.g. GitHub). See the Nature Portfolio guidelines for submitting code & software for further information.

Data

Policy information about availability of data

All manuscripts must include a data availability statement. This statement should provide the following information, where applicable:

- Accession codes, unique identifiers, or web links for publicly available datasets
- A description of any restrictions on data availability
- For clinical datasets or third party data, please ensure that the statement adheres to our policy

The mass spectrometry shotgun proteomics profiling data have been deposited to the ProteomeXchange Consortium via the PRIDE (Perez-Riverol et al., Nucleic Acids Res, 2019;47:D442-d450) partner repository with the dataset identifier PXD028842, and the SRM data have been deposited via PanoramaWeb and can be accessed via https://panoramaweb.org/neurochemistry_PD.url with the ProteomeXchange ID PXD028888.

Field-specific reporting

Please select the one below that is the best fit for your research. If you are not sure, read the appropriate sections before making your selection.

☒ Life sciences ☐ Behavioural & social sciences ☐ Ecological, evolutionary & environmental sciences

For a reference copy of the document with all sections, see nature.com/documents/nr-reporting-summary-flat.pdf

Life sciences study design

All studies must disclose on these points even when the disclosure is negative.

Sample size	No sample size calculation was performed, since this is an exploratory study. We included all PD and AP patients from whom cerebrospinal fluid was available from a previous longitudinal study. Limitations in sample availability are discussed in the manuscript.
Data exclusions	Data from 5 patients were excluded in the shotgun proteomics profiling part, due to changes in the clinical diagnosis of these patients (after a long-term follow-up) that were done after the proteomics profiling was performed. The diagnostic groups to be included were pre-defined.
Replication	The newly developed SRM method was extensively validated, including repeatability (intra- and inter-assay variation) and sample stability (on autosampler and freeze-thaw effects).
Randomization	Not applicable; this is not a treatment study.
Blinding	The investigators were not blinded to diagnosis at all steps of data collection, but all samples were measured in random order in all cases and deblinding did not affect the data analysis.

Reporting for specific materials, systems and methods

We require information from authors about some types of materials, experimental systems and methods used in many studies. Here, indicate whether each material, system or method listed is relevant to your study. If you are not sure if a list item applies to your research, read the appropriate section before selecting a response.

Materials & experimental systems

n/a	Involved in the study	
☒	☐	Antibodies
☒	☐	Eukaryotic cell lines
☒	☐	Palaeontology and archaeology
☒	☐	Animals and other organisms
☐	☒	Human research participants
☒	☐	Clinical data
☒	☐	Dual use research of concern

Methods

n/a	Involved in the study	
☒	☐	ChIP-seq
☒	☐	Flow cytometry
☒	☐	MRI-based neuroimaging

Human research participants

Policy information about studies involving human research participants

Population characteristics	Cerebrospinal fluid was used in the discovery study from in total 25 participants (PD, n = 10; MSA, n = 5; non-neurological controls, n = 10), and in the validation study from in total 110 participants (PD, n = 46; MSA, n = 17; PSP, n = 8; non-neurological controls, n = 39). Age and sex distribution was similar in both studies.
Recruitment	Participants from a previous longitudinal study were included based on clear clinical signs of parkinsonism, but with an yet unclear diagnosis at the time of inclusion. Participants had been recruited from our movement disorders outpatient clinic between January 2003 and December 2006 at the Radboud University Medical Center (Nijmegen, the Netherlands).
Ethics oversight	Use of cerebrospinal fluid was in this study approved by the local Medical Ethics Committee (Arnhem-Nijmegen; file no. 2002/188).

Note that full information on the approval of the study protocol must also be provided in the manuscript.
